# Adherence to 2020 US Multi-Society Task Force Guidelines for Post-Polypectomy Surveillance: a Multicenter Real-Time Analysis

**DOI:** 10.1007/s12029-026-01486-y

**Published:** 2026-06-20

**Authors:** Kacey Idouchi, Wasif Hassan, Mathew J. Gregoski, Olga C. Aroniadis, Don C. Rockey

**Affiliations:** 1 Digestive Disease Research Center, Medical University of South Carolina, 96 Jonathan Lucas Street, Clinical Sciences Building, Suite 908, Charleston, SC 29425 USA; 2 Department of Public Health Sciences, Medical University of South Carolina, Charleston, SC 29425 USA; 3 Division of Gastroenterology, Department of Internal Medicine, Stony Brook Medicine, Stony Brook, NY 11794 USA

**Keywords:** Colon, Polyp, Cancer, Screening, Quality, Endoscopy, U.S. Multi-Society Task Force

## Abstract

**Purpose:**

The U.S. Multi-Society Task Force (USMSTF) updated its 2020 guidelines to recommend less frequent post-polypectomy surveillance, yet premature colonoscopies remain common. Since the guidelines were published 5 years ago, we hypothesized that adherence has improved over time. We also aim to identify factors associated with non-adherence.

**Methods:**

This was a multicenter retrospective cohort study of 452 patients. 1st surveillance colonoscopies performed between 2020 and 2025 were included. Findings from pathology reports for both index and first surveillance colonoscopies were recorded. We considered follow-up after identifying hyperplastic polyps (HPs), low-risk adenomas (LRAs), or high-risk adenomas (HRAs) (the most advanced lesion was considered to be the lesion for which surveillance was indicated). Procedures performed more than 6 months earlier or later than recommended were defined as early or late, respectively.

**Results:**

Early surveillance occurred in 58% (*n* = 262/452) of 1st surveillance colonoscopies, while late surveillance occurred in 13% (*n* = 59/452). Early surveillance was performed in 82% of patients with only hyperplastic polyps (OR 5.47, 95% CI 1.60-18.72), and 64% of patients with only LRAs (OR 1.54, 95% CI 1.02–2.34). In contrast, patients with HRAs had delayed surveillance (OR 0.096, 95% CI 0.055–0.167). Early rates by year (2020–2025) were 46% (20/44), 50% (30/60), 57% (35/61), 70% (37/53), 51% (34/67), and 60% (100/167), respectively.

**Conclusions:**

Five years after the release of the 2020 USMSTF guidelines, adherence to recommended colonoscopy follow-up surveillance guidelines remains suboptimal. These findings highlight persistent gaps in guideline implementation.

## Introduction

Colorectal cancer (CRC) is one of the most common malignancies worldwide and remains a leading cause of cancer-related mortality, accounting for roughly 10% of all cancer cases and representing the second-highest number of cancer deaths globally [1]. Screening colonoscopy with polypectomy serves as the gold standard in preventing CRC by detecting and removing precancerous polyps [2–4]. Accordingly, optimizing the timing of surveillance colonoscopy is essential to balance cancer prevention with procedure-related risks, resource utilization, and cost.

Despite having established guidelines, adherence to recommended surveillance intervals remains highly variable. Prior studies have shown that overuse has been reported in ~ 16% of patients with no adenomas and 26% with low-risk adenomas (LRA) [5]. A 2004 survey of U.S. gastroenterologists and surgeons found that many clinicians had recommended a 3-year interval or shorter for small adenomas [6]. Community-based data examining a cohort of 3,876 individuals across nine U.S. communities similarly demonstrated over-utilization of surveillance colonoscopy among low-risk subjects and under-utilization among subjects with advanced adenoma [7–9]. Early surveillances and overutilization have been the main problem; thus, the USMSTF guidelines were updated to help with scope burden [10, 11]. One study found that adherence to colonoscopy surveillance guidelines would reduce the burden of surveillance of colonoscopy by 33–39%, especially during COVID-19 [1, 12]. Starting in 2020, the U.S. Multi-Society Task Force (USMSTF), on CRC, released updated surveillance recommendations designed to refine interval timing based on the number, size, and histology of detected polyps. Notably, the updated guidelines extended the surveillance interval from 5 to 10 years to 7–10 years for patients with 1–2 tubular adenomas < 10 mm. Prior work has proposed that these individuals may actually not need any surveillance until 10 years after baseline for LRA [13]. Some other changes to the USMSTF guidelines also include new specific recommendations for the timing of second surveillance colonoscopies based on both baseline and first surveillance findings for tubular adenomas. A retrospective cohort study showed that patients with 1–2 small tubular adenomas on screening colonoscopy and no adenomas on first surveillance still had an elevated prevalence of adenomas and advanced lesions on second surveillance, highlighting the importance of continued surveillance [14].

Overall, real-world data on adherence to the 2020 USMSTF guideline-recommended intervals for surveillance remain limited. Here, we hypothesized that adherence to the 2020 USMSTF guidelines has improved over time, and thus, we aimed to study risk factors related to premature and late surveillance.

## Methods

### Study Population and Design

This was a retrospective multicenter observational analysis of patients who had their 1 st surveillance colonoscopy in real-time. Index colonoscopies between 2012 and 2025 were included. Surveillance timing was evaluated according to the 2020 USMSTF recommendations for all first surveillance colonoscopies performed during the study period, Patient data were collected at the Medical University of South Carolina (MUSC) (Charleston, SC) and Stony Brook University Hospital (SBU) (Stony Brook, NY), both tertiary care academic medical centers, from March 2012 to July 2025. Patients were collected on ProVation who had indications written as “screening” and “surveillance”. Then on the EMR (Epic and Cerner), subsequent surveillance colonoscopies were recorded. Patients had to have had their index, first surveillance. If patients had their 2nd surveillance in real-time, it was also recorded. Colonoscopy findings and pathology reports were used to confirm the polyps. Findings on index and 1 st surveillance colonoscopy were collected, including examination date and polyp finds (number, size, and histology). The adenomas were then classified as low-risk adenoma (LRA), high-risk adenoma (HRA), sessile serrated polyps (SSP), hyperplastic polyps (HP), or no polyps. LRAs were defined if a patient had 1–2 tubular adenomas, each < 10 mm in size, and had low-grade dysplasia. HRAs were defined as ≥ 3 adenomas of any size, any adenomas ≥ 10 mm in size, any adenomas with villous or tubulovillous histology, and or any adenoma with high-grade dysplasia [15]. If HPs were found together with an SSP or TA, the case would be listed as SSP or TA, depending on which had the earliest recommendation given.

Patients at average risk who were ≥ 50 years old according to the 2012 guidelines, and ≥ 45 years old under the updated 2020 recommendations, were included to account for the revised CRC screening age. Normal screening colonoscopies were also included. Any patient having an inadequate bowel preparation (i.e., other than “adequate”, “good”, or “excellent”), unknown histology of previous polyps, or who were over the age of 75 were excluded. Patients with a history of heritable cancer syndromes (i.e., Lynch and Familial adenomatous polyposis), inflammatory bowel disease, history of CRC, or family history of CRC were also excluded.

Data were obtained from patient medical records. These data included demographic information (age at index colonoscopy, gender, and race). Demographic data was based on when the patient got their 1 st surveillance colonoscopy. This study was approved by the IRB at the Medical University of South Carolina (Protocol #00116200) and Stony Brook University Hospital (Protocol #202500238).

### Determining Early and Late Surveillance

This study aimed to assess the frequency of early and late surveillance colonoscopies and evaluate potential predictors such as adenoma type, endoscopist background, and bowel preparation quality. Recommendations were recorded based on messages or letters sent to the patient for their next interval colonoscopy. Patients were grouped into early, appropriate, and late based on index and 1 st surveillance polyp findings and compared them to the 2020 USMSTF guidelines. Colonoscopies were classified as *early* if performed more than six months before, or *late* if performed more than six months after, the recommended interval. Late surveillance was also considered as non-compliant as there was a 60% greater chance of finding adenomas as compared to patients getting their colonoscopies on time [16, 17]. Late colonoscopies in multiple studies have shown to have an increased risk for HRA or CRC [17–19].

### Determination for Open-Access Versus Gastroenterologist-Directed

Colonoscopy referrals or orders placed by a primary care physician were considered open access. Colonoscopies that were directly ordered by their gastroenterologists or that were seen in the gastroenterology clinic within the year were considered gastroenterologist-directed [20].

### Statistical Analysis

Descriptive statistics were reported as counts and percentages. Categorical variables were compared using Fisher’s exact test to evaluate differences in adherence rates. For analyses involving more than two subgroups, Bonferroni-adjusted *p*-values were applied to account for multiple comparisons among early, appropriate, and late surveillance groups.

To identify independent predictors of premature surveillance colonoscopies, univariate analyses were first performed. Each outcome was regressed individually on the following covariates: age, gender, race, African American ethnicity, polyp type, adenoma risk category, open access, and endoscopist experience. Variables with *p* ≤ 0.20 in univariate analyses were included in a multivariable logistic regression model using forward stepwise selection. Statistical significance was defined as *p* < 0.05. All analyses were conducted using SPSS Statistics, version 28 (IBM Corp., Armonk, NY, USA).

## Results

### Patients

**Fig. 1:**
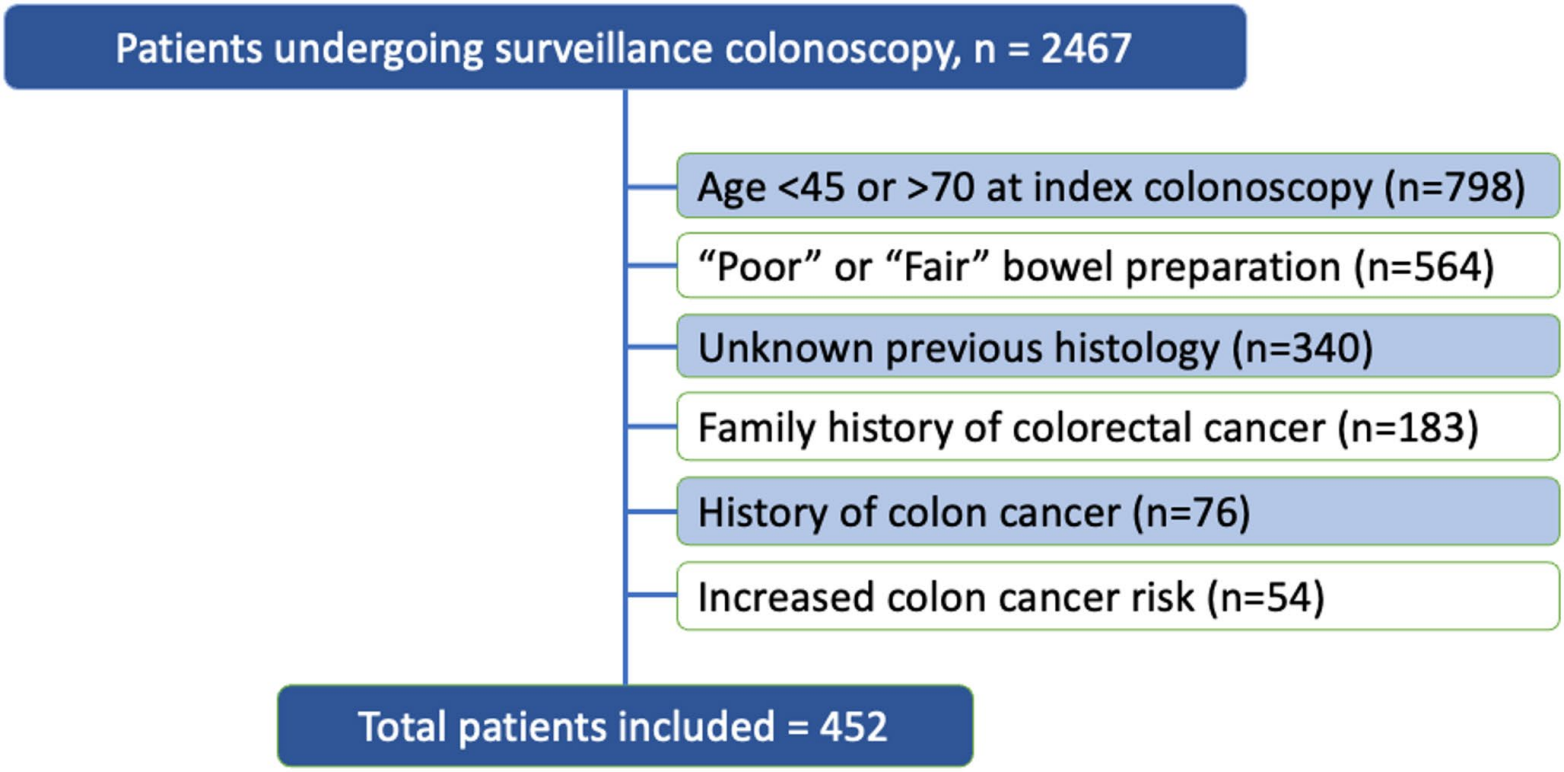
Patients. Shown is a flow diagram of patients included in the study

A total of 2,467 patients were evaluated, of whom 452 met the inclusion criteria and had screening, first surveillance colonoscopies done in real-time (Fig. 1). Of these, 316 patients were from MUSC and 136 from SBU. A total of 23 endoscopists, ranging from assistant professor to professor, performed the colonoscopies. The average age of the patients was 65 ± 7 with 49% (*n* = 221) being female (Table 1). The cohort included an equal number of men and women and was racially diverse. In terms of types of polyps identified, there were more patients with low-risk than high-risk adenomas (as defined in Methods).

**Table 1:** Demographic and clinical data

Variable	Total patients (*N* = 452)
Mean age (years)	65.35 (SD 6.7)
Gender	
Male	51.1% (231)
Female	48.9% (221)
Race/ethnicity	
White (non-Hispanic)	64.6% (292)
Black	32.1% (145)
Hispanic	0.4% (2)
Asian	1.5% (7)
Other	1.3% (6)
Method of Access	
Gastroenterologist directed	44.3% (139)
Open access	55.7% (175)
Polyp Types	
Low-risk adenoma	33.0% (149)
High-risk adenoma	23.5% (106)
Hyperplastic polyps	8.4% (38)
Sessile serrated adenoma	6.0% (27)

### Adherence Rates

Among the 452 patients included in the study, 131 (29%) had timing of colonoscopy that was adherent to guideline recommendations, 59 (13%) underwent late surveillance, and 262 (58%) had premature surveillance. Female patients were significantly more likely to undergo premature surveillance compared with males (146/221, 66% vs. 116/231, 50%; *p* < 0.003 after Bonferroni adjustment). Among polyp types, HPs had the highest premature rates at 82% (31/38), followed by SSPs at 52% (14/27) and tubular adenomas at 45% (115/255). Normal 1 st surveillances with no polyps found, had a premature rate of 77% (102/132). For LRA and HRA, LRA had markedly higher premature rates 64% (95/149) than high-risk adenomas (HRA) 19% (20/106) (*p* < 0.001). Race and ethnicity were not significantly associated with the likelihood of appropriate adherence (*p* = 0.195), and bowel preparation quality (*p* = 0.270) was also not significantly associated with appropriate adherence (Table 2). Endoscopist characteristics, including gender (*p* = 0.518), years of experience (*p* = 0.397), and age (*p* = 0.808) were also not associated with appropriate adherence.

**Table 2:** Surveillance outcomes

Variable	Adherent	Late	Early	*P*-value
Gender				
Male (*n* = 231)	79 (34%)	36 (16%^)^	116 (50%)	0.003
Female (*n* = 221)	52 (24%)	23 (10%)	146 (66%)	
Race/ethnicity				
White (non-Hispanic) (*n* = 292)	93 (32%)	33 (11%)	166 (57%)	0.195
Black (*n* = 145)	32 (22%)	26 (18%)	87 (60%)	
Hispanic, Asian, and other (*n* = 15)	6 (40%)	0 (0%)	9 (60%)	
Method of access				
Gastroenterologist directed (*n* = 139)	35 (25%)	27 (19%)	77 (56%)	0.656
Open access (*n* = 175)	47 (27%)	27 (15%)	101 (58%)	
Bowel prep				
Excellent (*n* = 98)	28 (29%)	11 (11%)	59 (60%)	0.270
Good (*n* = 255)	80 (32%)	29 (11%)	146 (57%)	
Adequate (*n* = 99)	23 (23%)	19 (19%)	57 (58%)	
Polyp type				
Tubular adenoma (*n* = 255)	92 (36%)	48 (19%)	115 (45%)	< 0.001
Hyperplastic polyp (*n* = 38)	7 (18%)	0 (0%)	31 (82%)	
Sessile serrated polyp (*n* = 27)	8 (30%)	5 (19%)	14 (51%)	
No polyps (*n* = 132)	24 (18%)	6 (5%)	102 (77%)	
Adenoma risk				
Low (*n* = 149)	39 (26%)	15 (10%)	95 (64%)	< 0.001
High (*n* = 106)	53 (50%)	33 (31%)	20 (19%)	
Endoscopist gender				
Female (*n* = 112)	33 (29%)	11 (10%)	68 (61%)	0.518
Male (*n* = 340)	98 (29%)	48 (14%)	194 (57%)	
Endoscopist experience				
<5 years (*n* = 2)	2 (100%)	0 (0%)	0 (0%)	0.397
5–10 years (*n* = 172)	50 (29%)	21 (12%)	101 (59%)	
>10 years (*n* = 278)	79 (28%)	38 (14%)	161 (58%)	
Endoscopist age				
40–46 years (*n* = 184)	55 (30%)	21 (11%)	108 (59%)	0.808
47–59 years (*n* = 144)	44 (31%)	19 (13%)	81 (56%)	
60 + years (*n* = 124)	32 (26%)	19 (15%)	73 (59%)	

Risk factors independently associated with early surveillance were identified by multiple logistic regression. Female sex was independently associated with increased odds of earlier surveillance (Adjusted OR 1.74, 95% CI 1.14–2.66, *P* = 0.011). LRA cases (Adjusted OR 1.54, 95% CI 1.02–2.34, *P* = 0.04) showed a higher risk for early colonoscopy but when a multiple logistic regression was performed, it was non-significant (Adjusted OR 0.65, 95% CI 0.41–1.05, *P* = 0.077). Factors such as age, race, African American ethnicity, open access, and endoscopist experience were not significant for forward selection as shown in Table 3.

**Table 3:** Risk factors associated with premature surveillance

Variable	Univariate Analysis	
	*Adjusted OR (95% CI)	*P*-value
Age	0.99 (0.96–1.02)	0.44
Female gender	1.93 (1.32–2.82)	< 0.001
Race	1.08 (0.81–1.43)	0.62
African American	1.132 (0.67–1.91)	0.64
Open access	0.908 (0.57–1.43)	0.68
HP	5.47 (1.60–18.72.60.72)	0.007
HRA	0.096 (0.055–0.167)	< 0.001
LRA	1.54 (1.02–2.34)	0.04
Endoscopist experience	1.054 (0.711–1.56)	0.794

*An OR > 1 indicates higher odds of early surveillance, whereas an OR < 1 indicates lower odds of early surveillance

When looking into late surveillances, HRAs were more associated with late colonoscopies at a rate of 31.1% (33/73). HRAs (OR 0.08, 95% CI 0.04–0.14, *P* = 0.001) were more likely to have timely or delayed surveillance. We also looked into physician-recommended intervals for tubular adenomas that were documented in patient letters. Adherence rate was 50.2% (128/255) between index and 1 st surveillance, and 35.7% (91/255**)** between 1 st and 2nd surveillance colonoscopies in tubular adenomas. We were not able to look at 2nd surveillances in real time.

### Open-Access Versus Gastroenterologist-directed Premature Surveillance

There were 23 gastroenterologists in the GI-directed group and 52 primary care providers, including physician assistants and nurse practitioners, in the open access group. Early rates showed no significant difference between GI-directed and open-access (55% (77/112) vs. 58% (101/148), *p* = 0.656).

### Changes in Adherence Over the Years

Early surveillance rates (as described in the Methods - measured by whether 1 st surveillances were done on time according to guideline-recommended intervals) by year (2020–2025) were 46% (20/44), 50% (30/60), 57% (35/61), 70% (37/53), 51% (34/67), 60% (100/167). The mean interval for premature 1 st surveillance colonoscopies was 40 ± 21 months, while late 1 st surveillance colonoscopies were delayed by 17 ± 14 months.

## Discussion

It has been several years since the new 2020 USMSTF guidelines were released. Here, we have shown that despite the time that has lapsed since 2020, premature rates of surveillance colonoscopy after detection of HPs and LRAs remain high - with an 82% and 64% rate of premature colonoscopy, respectively. In contrast, it was notable that patients with HRAs had a late colonoscopy, with a rate of 31% (33/106). These data raise a number of important points.

Adherence with USMSTF surveillance guidelines has been reported to be low after screening colonoscopy for at least the last decade [1, 10, 21]. In one study, 36% of physician recommendations were non-adherent to the 2012 guidelines [5]. In another study, non-adherence rates were 28% after normal colonoscopies, 52% after identification of HPs, 45% after finding LRAs, and 49% after HRAs [22]. It has been postulated that endoscopists were slow to adopt or disagreed with the new guidelines [1, 21]. The biggest change to the new guidelines was an increase in the interval for repeat colonoscopy in patients with 1–2 small adenomas (from 5 to 10 years to 7–10 years). Other changes included increasing the interval for patients with 3–4 small adenomas from 3 to a range of 3–5 years and new specific recommendations for the timing of second surveillance colonoscopies based on both baseline and first surveillance findings for tubular adenomas [8, 23]. When looking into physician-recommended interval recommendations after having their first surveillance we saw that the adherence rate was 50.2% between index and 1 st surveillance, and 35.7% between 1 st and 2nd surveillance colonoscopies in tubular adenomas. This could be due to endoscopists not being aware that there are interval recommendations for baseline 1 st surveillance findings, or it could also be due to the complexity of looking back at the baseline screening colonoscopy findings. Interestingly, during the time frame after publication of the 2012 USMSTF guidelines, more than half of gastroenterologists stated that for a single small adenoma, they would recommend an interval of 3 years, whereas the new recommendations are for repeat colonoscopy after at least 5 years [9, 24]. For higher-risk adenomas, gastroenterologists have historically recommended frequent follow-up (3 years or sooner in a 2004 national survey) [6]. These persistent discrepancies between guideline recommendations and real-world practice underscore the need to better understand factors driving premature surveillance.

Our findings are in part consistent with previous data and in addition add additional information to the field. In a study that examined compliance with the 2020 guidelines after an initial screening colonoscopy, adherence to the guidelines was 49% for all polyps, ranging from 8% for LRAs to 88% for HRAs, 89% for HPs, to 63% for SSPs [10]. While the overall compliance rate in this study and the current study was similar, the rate for HP and HRA in our study was notably different. We observed higher rates of premature surveillance in HPs and delayed surveillance in HRAs. Notably, the prior study did not examine patterns of premature versus delayed surveillance or evaluate other patient- and procedure-level factors associated with these outcomes. In addition, that analysis was performed approximately two years after guideline release, whereas our study assessed adherence over a longer post-implementation period, allowing evaluation of temporal trends in real-world practice. It has been postulated that maybe endoscopists are slow to adopt or disagree with new guidelines [1, 21]. A prior study of 10 endoscopists found that they all selected the correct recommendation in a surveillance vignette with one tubular adenoma, and 7 endoscopists did so for a vignette with two tubular adenomas. It demonstrated that endoscopists were aware of the 2020 guidelines, with LRA recommendations continuing to show the poorest compliance [10].

Factors contributing to low adherence rates have been hypothesized to include poor bowel preparation quality, overestimation of cancer risk in adenomatous or hyperplastic polyps, and the presence of multiple comorbidities [22, 25]. A prior study suggested that inadequate bowel preparation only contributed to 17% of premature cases, which is consistent with the finding in our study that bowel preparation was not independently associated with late or early surveillance [20]. Other studies have found that endoscopists who completed training more than 10 years ago or who performed high volumes of colonoscopies were more likely to recommend premature surveillance colonoscopies [10]. In the current study, se excluded patients with poor or equivocal bowel preparation, so this is unlikely to be a factor. We examined other factors such as race, open access vs. gastroenterologist-directed, endoscopist gender, endoscopist experience, and endoscopist age – and found that none of these had a significant association with inappropriate early colonoscopy. Interestingly, female patients were found to be most likely to undergo early colonoscopy. A prior study found that younger age, female sex, and the presence of gastrointestinal symptoms—including abdominal pain and diarrhea—were associated with inappropriate colonoscopy utilization [26].

One of the notable findings of this study was that we found no improvement in 1 st surveillance compliance rates from 2020 to 2025 after the publication of the 2020 recommendations. For the earlier years (e.g. 2020, 2021), it could be argued that endoscopists were slow to adopt updated guidelines; however, in another study, patients with LRAs still had low compliance rates even among endoscopists who correctly identified the 2020 USMSTF recommendations in a survey study [10], suggesting that low compliance could not be fully explained by low guideline awareness. 2020 was also the start of the COVID-19 pandemic, where 33% of patients’ colonoscopies were appropriate for rescheduling in a future year, according to the 2020 USMSTF guidelines [1]. During the pandemic, this was the best time to delay colonoscopies as there was a need to conserve healthcare resources [1]. In this scenario, it seems that the rate of early 1 st surveillance should be lower and thus, compliance rates should be higher. Thus, the theory that endoscopists are slow to adapt to the guidelines may not be correct.

Several strategies have been proposed to improve compliance. One approach is to optimize the EMR to incorporate guideline-based recommendations at two key points of care: immediately after colonoscopy and within primary care, using data already available within the EMR [1]. Automated algorithms – including AI tools - can help auto-populate polyp findings and recommended intervals, thus reducing variability in documentation. Additionally, patient and clinician-directed reminders, including mailed letters, text messages, or portal notifications, may further improve adherence by increasing patient engagement and reducing missed or delayed follow-up [27]. Other proposed solutions include standardized endoscopy report templates to minimize ambiguity in surveillance recommendations, audit-and-feedback programs to provide endoscopists with individualized adherence metrics, and institutional protocols that require verification of prior colonoscopy data before scheduling surveillance exams [28–30].

We recognize the strengths and limitations of this study. Importantly, this study was performed at two academic tertiary medical centers, which enhances its generalizability. However, because these are academic medical centers, the findings may not be generalizable to other institutions, such as community hospitals. Our study further examines patterns of premature versus delayed surveillance in relation to the 2020 USMSTF guidelines and evaluates temporal trends following guideline adoption. Some other limitations are that patients older than 75 years were excluded from our analysis, which restricts our ability to interpret surveillance practices in this population, for whom clinical decision-making remains controversial [31]. The sample size was also limited for HPs and SSPs. This was due to a paucity, as expected, of patients who had only hyperplastic polyps (HPs). Additionally, reasons for late and early colonoscopies were not tracked, so alternative explanations for early and late colonoscopies could not be explored. Finally, we also did not assess gastroenterologists’ awareness of the updated guidelines, despite the increased time available for practice adaptation.

In summary, we have shown here that there is a pervasive lack of adherence to 1 st surveillance guidelines for follow-up colonoscopy. Surprisingly, the propensity for this lack of adherence did not improve over a 5-year period after publication of updated 2020 USMSTF guidelines. The data suggest that as a result of early surveillance colonoscopy, substantial endoscopic resources are being allocated to low-risk individuals—such as those with hyperplastic polyps or small tubular adenomas—who derive limited clinical benefit yet contribute significantly to resource utilization. We speculate that novel approaches to guideline implementation are needed.

## Data Availability

No datasets were generated or analysed during the current study.

## Abbreviations

CRC: colorectal cancer
HRA: high-risk adenoma
LRA: low-risk adenoma
USMSTF: U.S. Multi-Society Task Force guidelines
SSP: sessile serrated polyp
HP: hyperplastic polyp
